# A High-Density SNP Genetic Map Construction Using ddRAD-Seq and Mapping of Capsule Shattering Trait in Sesame

**DOI:** 10.3389/fpls.2021.679659

**Published:** 2021-06-01

**Authors:** Engin Yol, Merve Basak, Sibel Kızıl, Stuart James Lucas, Bulent Uzun

**Affiliations:** ^1^Department of Field Crops, Faculty of Agriculture, Akdeniz University, Antalya, Turkey; ^2^Department of Medicinal and Aromatic Plants, Akev University, Antalya, Turkey; ^3^Sabanci University Nanotechnology Research and Application Center (SUNUM), Istanbul, Turkey

**Keywords:** neglected crop, annotation, breeding, high throughput sequencing, linkage map

## Abstract

The seed-bearing capsule of sesame shatters at harvest. This wildish trait makes the crop unsuitable for mechanized harvesting and also restricts its commercial potential by limiting the cultivation for countries that have no access to low-cost labor. Therefore, the underlying genetic basis of the capsule shattering trait is highly important in order to develop mechanization-ready varieties for sustainable sesame farming. In the present study, we generated a sesame F_2_ population derived from a cross between a capsule shattering cultivar (Muganli-57) and a non-shattering mutant (PI 599446), which was used to construct a genetic map based on double-digest restriction-site-associated DNA sequencing. The resulting high-density genetic map contained 782 single-nucleotide polymorphisms (SNPs) and spanned a length of 697.3 cM, with an average marker interval of 0.89 cM. Based on the reference genome, the capsule shattering trait was mapped onto SNP marker S8_5062843 (78.9 cM) near the distal end of LG8 (chromosome 8). In order to reveal genes potentially controlling the shattering trait, the marker region (S8_5062843) was examined, and a candidate gene including six CDSs was identified. Annotation showed that the gene encodes a protein with 440 amino acids, sharing ∼99% homology with transcription repressor KAN1. Compared with the capsule shattering allele, the SNP change and altered splicing in the flanking region of S8_5062843 caused a frameshift mutation in the mRNA, resulting in the loss of function of this gene in the mutant parent and thus in non-shattering capsules and leaf curling. With the use of genomic data, InDel and CAPS markers were developed to differentiate shattering and non-shattering capsule genotypes in marker-assisted selection studies. The obtained results in the study can be beneficial in breeding programs to improve the shattering trait and enhance sesame productivity.

## Introduction

Sesame (*Sesamum indicum* L.) is an important oilseed crop which belongs to the *Sesamum* genus of the Pedaliaceae family. It is cultivated in more than 60 countries in an area of 11.74 m ha, with a total production of 6.0 m tons (FAO, 2018). Although the production quantity is much lower than major oilseeds such as sunflower, rapeseed, and groundnut, the total production of sesame seed has increased by about 40% in the last decade (FAO, 2018). The high demand for this crop is related to its high-quality vegetable oil (Arslan et al., 2007; Uzun et al., 2008) and antioxidant lignans, including sesamin and sesamolin (Yoshida and Takagi, 1997; Moazzami and Kamal-Eldin, 2006). Its seeds have long been used in human nutrition, cosmetics, perfumery, soaps, insecticides, anti-aging treatments, and medicine. The health benefits of sesame have also been reported to reduce the rate of occurrence of certain cancers (Miyahara et al., 2001), constrain the growth of leukemia cells (Ryu et al., 2003), and decrease the susceptibility of low-density lipoprotein (LDL) to oxidation, which is recognized as a risk factor for atherosclerosis (Nakamura et al., 2020). In addition, sesame has agricultural advantages; it can survive in extreme conditions, can grow on only soil moisture without irrigation and fertilizer, and can be grown as a second crop (Ashri, 2007). These attributes make the crop an important income source especially for small-scale farmers in developing countries (Dossa et al., 2017).

Despite its agricultural, nutritional, and economic importance, the yield of sesame is lower than those of other major oilseeds (Hwang, 2005). It has some wildish traits, namely, capsule shattering (Uzun et al., 2004), indeterminate growth habit (Uzun and Cagirgan, 2006), and non-synchronous maturity, which are the main causes of low seed yield. Capsule shattering is the most serious problem in sesame production, as it causes seed losses at harvest that can be very high, up to 50% (Weiss, 1971). This wildish trait also restricts sesame’s commercial potential and limits its economic viability in countries that have no access to cheap labor because it makes the crop unsuitable for mechanized harvesting (Langham and Wiemers, 2002). Therefore, non-shattering and/or semi-shattering capsule genotypes should be useful for adapting sesame to mechanized harvesting.

A closed (non-shattered) capsule mutant trait was discovered by Langham (1946); however, over 99% of the sesame used for production in the world still has the shattering trait (Langham, 2001). Unwanted side effects such as cupped leaves, short seed pods, twisted stems, and low seed yield restrict the direct use of this non-shattering mutant type (Wongyai et al., 2001). Out-crosses between closed capsule mutants and advanced cultivars have been conducted in order to eliminate these side effects and also improve traits of agricultural importance (Uzun et al., 2004; Yol and Uzun, 2019). Although heterotic improvements in performance were obtained for seed yield and yield-related traits in these studies, there was no progress with respect to suitability for mechanized harvesting. Besides conventional breeding techniques, molecular markers have also been developed for the closed capsule mutant trait for use in marker-assisted selection (Uzun et al., 2003; Phumichai et al., 2017; Zhang H.Y. et al., 2018). However, there is a need to understand the mechanism of the shattering trait at the genomic level in order to increase the efficiency and efficacy of breeding studies.

High-throughput, “next-generation” sequencing technologies provide opportunity for the development of high-density maps to identify genes and quantitative trait loci (QTLs) for agronomically important traits (Nguyen et al., 2019). Especially reduced representation sequencing (RRS) approaches make low-cost and flexible genotyping in the target genomes possible (Scheben et al., 2017). Double-digest RAD sequencing (ddRAD-Seq) (Peterson et al., 2012) is one of the RRS methods for SNP discovery at a genome-wide scale. It includes digesting the DNA with selected restriction enzymes and then sequencing a specific size-selected range of generated fragments (Aguirre et al., 2019). It eliminates the shearing step of the original RAD protocol (Baird et al., 2008) and also provides more flexibility with respect to the targeted marker density and the number of fragments (Robledo et al., 2018) compared to RAD-Seq (Baird et al., 2008) and/or 2b-RAD (Wang et al., 2012). The ddRAD-Seq protocol has several advantages: the degree of genome coverage can be adjusted by the choice of different restriction enzymes, high efficiency and accuracy are provided in SNP discovery, and no reference genome is required in the bioinformatic process (Peterson et al., 2012). It has been used for high-density genetic map construction in strawberry (Davik et al., 2015), northern red oak (Konar et al., 2017), and platycladus (Jin et al., 2019). QTL mapping has been used for different traits in rapeseed (Chen et al., 2017), lettuce (Seki et al., 2020), and peanut (Liang et al., 2021) and in genetic diversity studies in orchid (Roy et al., 2017), onion (Lee et al., 2018), and sesame (Basak et al., 2019). However, to the best of our knowledge, there is no report about the construction of a genetic map in sesame with the use of SNPs discovered by ddRAD-Seq technology.

In this study, the F_2_ population that was developed by a cross between a capsule shattering sesame cultivar × non-shattering mutant line was sequenced by ddRAD-Seq. This genotypic data was used with the following aims: (i) to construct a genetic linkage map with SNPs, (ii) to reveal the genomic basis of the capsule shattering trait, and (iii) to develop markers for this trait. These findings will contribute to new breeding strategies in sesame in order to develop high-yielding genotypes that are also suitable for mechanized harvesting.

## Materials and Methods

### Plant Material and F_2_ Population Development

**FIGURE 1:**
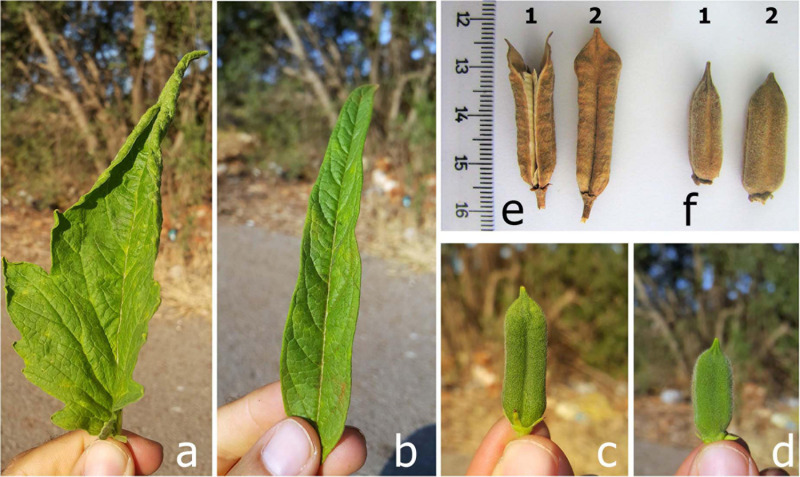
The morphological differences for capsule shattering and non-shattering genotypes. **(a,d,f)** Leaf, capsule-forming stage, and harvested capsule of the non-shattered capsule phenotype, respectively. **(b,c,e)** Leaf, capsule-forming stage, and harvested capsule of the capsule shattering phenotype, respectively.

The F_2_ mapping population comprised of 120 individuals derived from a cross between a sesame cultivar (Muganli-57) and a sesame line. The male parent, Muganli-57, was registered by the West Mediterranean Agricultural Research Institute, Turkey. It has high seed yield, yellow seeds, about 50% oil content, and shattered capsules. The female parent (mutant line) was introduced from USDA GenBank with the accession number PI 599446, named Paloma. It is distinct from the cultivar in its agro-morphological traits. This line had low seed yield, brown seed color, curly leaves, short, round shape, and non-shattered capsules. Figure 1 shows the morphological differences between the leaf and capsules of these genetic materials.

The parents were crossed in the greenhouse of Akdeniz University (36°53′ N, 38°30′ E and altitude of 15 m) during the 2015 growing season in Antalya, Turkey. A total of 1,496 F_2_ seeds were harvested from self-pollinated F_1_ plants and grown in four rows with a row-to-row distance of 70 cm and plant-to-plant distance within a row of 10 cm at the same location in 2017. These lines were phenotypically scored and labeled according to leaf and capsule shapes as capsule shattering and non-shattering genotypes in the maturation stage. A chi-square goodness of fit test was performed on the F_2_ population to confirm the segregation ratio for the capsule shattering trait. A total of 120 F_2_ individuals, which included 90 shattering and 30 non-shattering types as well as both parents, were selected for sequencing and the construction of the genetic map.

### DNA Extraction and ddRAD-Seq

Healthy young leaves from 120 F_2_ individuals and the two parents were collected and stored at −80°C. Extraction of genomic DNA was carried out with a sodium dodecyl sulfate-based method (Ahmed et al., 2009) with minor modifications. DNA concentration and quality were measured with a Qubit 2.0 Fluorometer using a Qubit DNA Assay Kit (Life Technologies, Thermo Fisher Scientific).

A modified version of the ddRAD-Seq method (Peterson et al., 2012) was used for reduced representation genomic library construction. Briefly, the restriction enzymes *Vsp*I and *Msp*I were employed to digest the genomic DNA of each individual. Ampure XP beads (Beckman Coulter Genomics) were used to clean the digested products followed by P1 and P2 adapter ligation with T4 ligase buffer. The 3′ end of the P1 adapter was modified because six-base cutter *Vsp*I restriction enzyme was selected instead of *Eco*RI, which is the enzyme used in the original ddRAD protocol. Following ligation, genotype-specific indexed PCR primers were used in PCR amplification for each reaction. The amplified products were then checked on an agarose gel and pooled in equal concentration. Size selection (400–500 bp) was carried out on the pooled products in a final step. The reduced representation genomic libraries were sequenced using an Illumina HiSeq 2,500 instrument at Macrogen (Seoul, Republic of Korea) for 150-bp paired-end sequencing. The ddRAD sequence data for studied samples have been deposited with links to BioProject accession number PRJNA633276 in the NCBI.

### Bioinformatic Analysis for SNP Discovery

The bioinformatic pipeline was operated using the Galaxy^1^ which is open, web-based computational platform. In the first step of analysis, raw reads were demultiplexed to genotype-specific fastq files with Je (v1.2.1) (Girardot et al., 2016). All sequencing files were then processed to filter out low-quality reads using fastp (v0.20.1) (Chen et al., 2018). In this step, reads having an average phred-scaled quality score less than 15 and containing restriction enzyme sequences were trimmed. Bowtie2 (v2.3.4.3) (Langmead and Salzberg, 2012) was used to align the cleaned reads to the sesame reference genome “Zhongzhi13 v2.0” (Wang et al., 2016) with the default parameters. The created genotype-specific individual BAM (binary sequence alignment file format) files were analyzed in a variant calling program, freebayes (bayesian genetic variant detector) (v13.1) (Garrison and Marth, 2012), with the calling model for simple diploids and filtering to include only sites with a total read coverage value of at least 20×. Bcftools merge (v1.10) (Li et al., 2009) was used to combine each genotype-specific variant file (.vcf) into a single variant file which was then converted to browser extensible data (BED) format to store the genomic regions as coordinates and associated annotations. Afterward, individual BAMs were re-analyzed on freebayes with the BED dataset, retaining sequence differences with at least 6× coverage to reveal reliable variants for each genotype, as before the created .vcf files were combined to a single variant file using the merge option from bcftools. This merged file was then further filtered with Tassel V5.2.52 (Glaubitz et al., 2014) to detect polymorphic sites using these parameters: minimum site count 80 and minimum–maximum heterozygous proportion 0.3–0.8. The filtered file was then converted to ABH genotype format for the mapping analysis.

### Linkage Map Construction

Markers exhibiting missing data (> 20%) and segregation distortion (χ^2^-test, *P* < 0.001) were excluded. The remaining markers were used to construct a genetic linkage map with the JoinMap 4.1 program (Van Ooijen, 2011). Markers were grouped into linkage groups with a minimum logarithm of odds score of 10.0 and maximum recombination of 45%. The regression mapping algorithm was employed with the following parameters: goodness-of-fit jump threshold for removal of loci = 5.0, third round = yes and “ripple” was performed after adding each marker. The recombination rate was converted into map distance in cM (centimorgans) by the Kosambi mapping function (Kosambi, 1994). The capsule shattering trait was scored as a qualitative trait and analyzed with allelic data using JoinMap 4.1. The genetic linkage map was graphically visualized and modified in MapChart 2.32 (Voorrips, 2002).

### Prediction of Gene Function

The flanking sequences of the SNP markers in the candidate gene were identified from the sesame reference genome. The potential gene coding sequence was predicted with FGENESH^2^ (Solovyev et al., 2006) based on sesame genome-specific parameters. The predicted gene regions from the shattering and non-shattering genotypes were amplified and sequenced with the Sanger method. These sequences were re-analyzed in FGENESH, and then functional annotation of the candidate gene was carried out manually by comparison with known proteins using the blastp algorithm in NCBI.

### InDel and CAPS Marker Development

We designed cleaved amplified polymorphic sequence (CAPS) and insertion--deletion (InDel) markers associated with the capsule shattering trait. These were tested on two parents and F_2_ individuals. Primers were designed using Primer3 web, version 4.1.0^3^, for both markers. PCR was performed in a 20-μl reaction volume with 1 μl of × 10 PCR buffer, 2.5 mM MgCl2, 0.5 μl of dNTP mix, 0.4 μl each of forward and reverse primers (10 pmol), 0.3 μl of Taq DNA polymerase (5 U/μl), 1.5 μl of genomic DNA, and Milli-Q water. Thermocycling started at 94°C for 5 min, followed by 45 cycles of 30 s at 92°C, 45 s at 57°C, 180 s at 72°C, and finally kept at 72°C for 10 min. For the CAPS marker, the appropriate restriction enzyme for the selected SNP region was identified in NEBcutter V2.0^4^. After PCR amplification, the PCR product was restriction-digested in a total volume of 20 μl for 1 h according to the manufacturer’s protocol (New England Biolabs, MA, United States). Then, 10 μl of the products for CAPS and InDel markers was separated by electrophoresis on 2% agarose gel and visualized by UV light.

## Results

### Morphology and Inheritance of the Capsule Shattering Trait

The sesame cultivar, Muganli-57, had higher plant height and number of capsules per plant compared to the line, PI 599446 (Yol and Uzun, 2012). They also differ significantly from each other with respect to leaf and capsule phenotypes (Figure 1). Although the cultivar type had normal sesame plant architecture, the line showed curly and bigger leaves. Hairy and short seed pods (Figure 1) and high-density blooming were also observed in the line at maturity. Compared with Muganli-57, this type maintains a non-shattering capsule structure in harvest time (Figure 1). These visible phenotypes provide a clear difference between cultivar and PI 599446 in all growing period.

The seed pods of the F_1_ plants developed from the hybridization of the line (non-shattering capsule) with Muganli-57 were all completely shattered at maturity, indicating the dominance of the capsule shattering trait. To identify the genetic basis underlying the capsule shattering phenotype, a large population of 1,496 F_2_ individuals were planted in an experimental field. The segregation of shattered capsule to non-shattered capsule plants closely fitted the ratio of 3:1 (χ^2^ = 2.22, *p* > 0.05) (Table 1), suggesting that the capsule shattering trait is controlled by a single gene, in which the shattering allele is dominant over the non-shattering allele.

**TABLE 1:** Segregation ratio of capsule shattering trait in sesame.

**Cross**	**Observed**		**Expected**		**χ^2^**	***P***	**F_2_ ratio**
	**Shattered capsule genotype**	**Non-shattered capsule genotype**	**Shattered capsule genotype**	**Non-shattered capsule genotype**			
Mutant line × Muganli-57	1,097	399	1,122	374	2.22	0.136	3:1

### Genotyping

A total of 122 sampled individuals, that is 120 F_2_ progeny and two parents, were used in the construction of ddRAD-seq library. After sequencing, about 40.0 Gb of sequence reads was obtained. Two individuals, N6 and N12, were discarded from this data due to very low read numbers. The remaining raw data containing 488.32 M reads was filtered with quality parameters, and 468.17 M of clean data was obtained for further analysis (Supplementary Figure 1). The highest and lowest filtered read counts were 0.51 and 9.16 M for the lines N244 and N238, respectively, with the mean of 3.87 M reads per individual in the F_2_ population. The GC content ranged from 36.39 to 39.91% (Supplementary Figure 1). The mapping rates to the *S. indicum* L. reference genome for the shattered capsule cultivar (Muganli-57) and non-shattered capsule mutant line (PI 599446) were 78.6 and 80.41%, respectively, while that of the F_2_ population was 79.28% on average. A total of 19,457 SNPs were initially called from the F_2_ lines using the variant calling pipeline (see section “Materials and Methods”). The maximum and minimum average distance per SNP was identified as 10.7 and 17.6 kb on chromosomes 3 and 13, respectively, in the raw SNP data (Table 2). After filtering to select high-confidence polymorphic sites, a total of 1,040 polymorphic SNP markers were detected. Of these, 1,027 SNPs were distributed between all 13 sesame chromosomes, and the remaining 13 SNPs were unmapped. The highest number of SNPs was detected on chromosome 8 (165 SNPs), whereas the lowest number of SNPs was found on chromosome 11 (13 SNPs), with an overall mean of 79 SNPs per chromosome. Of the 1,040 polymorphic SNPs, more SNPs were of transition than transversion type, with the most frequent being 160 A/G and 158 C/T SNPs, accounting for 15.38 and 15.20%, respectively (Table 3).

**TABLE 2:** Distribution of single-nucleotide polymorphisms (SNPs) in each chromosome.

**Chromosome**	**Initial number of SNPs**	**Average map length per SNP (kb)**	**Filtered number of SNPs**
Entire genome	19,457	13.2	1,040
Chr 1	1,811	11.1	106
Chr 2	1,194	15.2	69
Chr 3	2,402	10.7	98
Chr 4	1,307	15.6	85
Chr 5	1,234	13.3	39
Chr 6	2,207	11.7	153
Chr 7	1,179	13.9	28
Chr 8	1,893	13.5	165
Chr 9	1,667	13.6	110
Chr 10	1,322	14.6	69
Chr 11	920	15.1	13
Chr 12	1,156	13.9	54
Chr 13	922	17.6	38
Scaffolds	243	–	13

**TABLE 3:** Single-nucleotide polymorphism statistics in filtered data showing the number of alleles and the rate of allele occurrence.

**Allele^*a*^**	**Number of alleles**	**Proportion of allele occurrence (%)**
A:G	160	15.38
C:T	158	15.20
T:C	152	14.62
G:A	122	11.73
A:T	73	7.02
T:A	73	7.02
T:G	57	5.48
C:A	53	5.10
G:T	53	5.10
G:C	51	4.90
A:C	50	4.80
C:G	38	3.65

*^*a*^The first and second letters are reference and alternate alleles.*

### Linkage Map Construction

**FIGURE 2:**
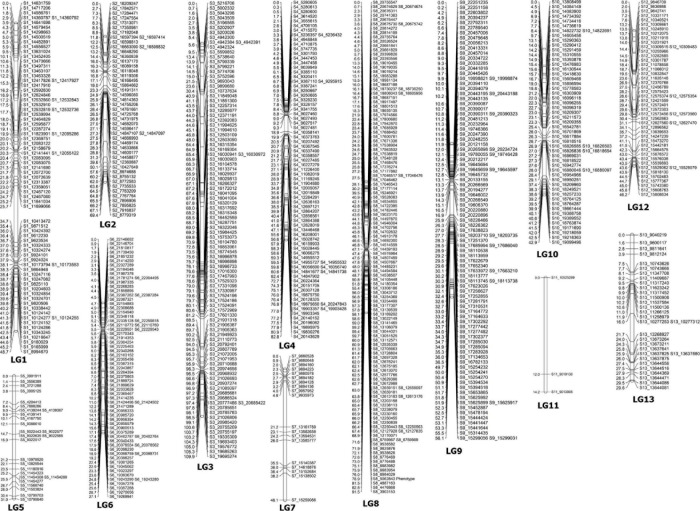
A genetic linkage map of sesame constructed using the F_2_ population of PI 599446 × Muganli-57.

Before the construction of linkage maps, SNP markers that did not fit the expected 1:2:1 segregation ratio or included high missing data were excluded from further analysis. In the linkage map, a total of 782 SNPs were assigned to 13 linkage groups (LGs) (Table 4 and Figure 2). This map covered a total genetic distance of 697.3 cM, with the LGs ranging from 14.2 to 109.9 cM, with an average marker interval of 0.89 cM. The LG with the greatest number of markers was LG8, which included 125 markers, with an average distance of 0.73 cM. Conversely, LG11 had the fewest markers, had the lowest marker density (4.73 cM), and was also the shortest LG. LG13, the longest LG, had a genetic length of 109.90 cM and contained 97 markers, with an average distance between markers of 1.13 cM. The highest marker density was observed in LG6 (an average of 0.38 cM between markers), followed by LG1 (0.62 cM) (Table 4); the average distance between markers was under 1 cM in seven LGs (Table 4). The largest gap was of 16.54 cM, located in LG7, followed by 16.46- and 13.24-cM gaps in LG3 and LG9, respectively.

**TABLE 4:** Basic characteristics of the 13 linkage groups.

**Linkage group**	**Number of markers**	**Total distance (cM)**	**Average distance (cM)**	**Maximum gap (cM)**
LG1	79	48.7	0.62	8.98
LG2	48	69.4	1.45	10.48
LG3	97	109.9	1.13	16.46
LG4	80	82.8	1.04	10.34
LG5	27	31.0	1.15	5.14
LG6	71	26.9	0.38	3.21
LG7	19	48.1	2.53	16.54
LG8	125	91.5	0.73	9.32
LG9	99	56.1	0.57	13.24
LG10	55	42.9	0.78	8.34
LG11	3	14.2	4.73	11.9
LG12	49	46.2	0.94	5.63
LG13	31	29.6	0.95	5.69
Total	783	697.3	0.89	16.54

### Mapping of Shattering Trait and Candidate Gene Prediction

**FIGURE 3:**
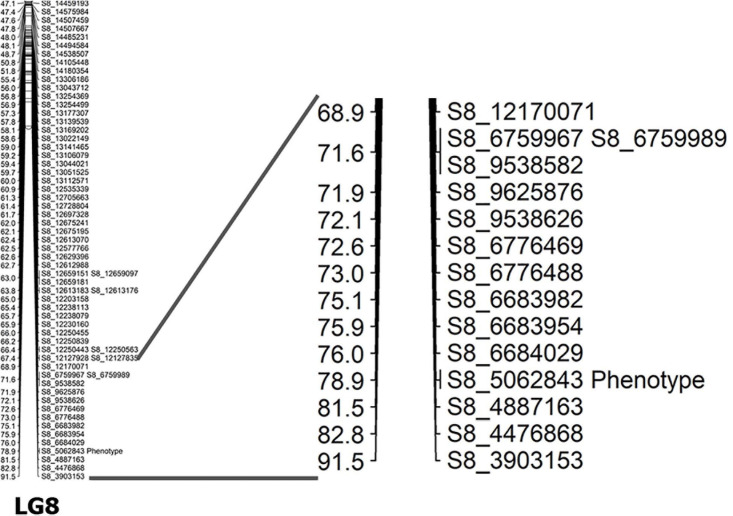
Detailed graphics for the position of the capsule shattering trait (phenotype) on LG8.

**FIGURE 4:**
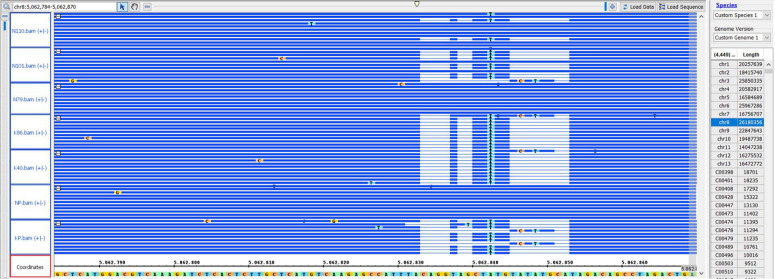
IGB shows the marker position of S8_5062843 for the selected genotypes. NP, shattered capsule parent; KP, non-shattering capsule parent. N110, N101, and N79 are F_2_ lines that had the shattered capsule trait. K40 and K86, non-shattering lines in F_2_. The coordinates show the reference genome sequence.

Capsule shattering is predicted to be controlled by a single gene, and this phenotype was scored as a qualitative trait in the mapping analysis. Based on the Zhongzhi13, v2.0, sesame reference genome, it was mapped to coincide with SNP marker S8_5062843 (78.9 cM) near the end of LG8 (chromosome 8) (Figure 3). The locus containing the SNP marker was visualized for capsule shattering and non-shattering parents and selected F_2_ lines (Figure 4). On chromosome 8, the nucleotide “A” was found in the reference genome at this SNP position, while the “T” nucleotide was observed in the non-shattering parent line (KP). Accordingly, the parental line (NP) with the shattered capsule trait had a sequence identical to the reference genome in the specified region. Heterozygosity was observed in some F_2_ lines such as N101 and N110 for this SNP, with individual reads matching both parental alleles; these lines had the shattered capsule phenotype. Other F_2_ lines such as K40, K86, N79, and N91 were homozygotes with the same alleles at corresponding chromosomal loci as their parents (Figure 4). When all F_2_ lines were examined, a 1:2:1 segregation ratio was obtained for this SNP (27:48:29, non-shattered capsule/heterozygous/shattered capsule) in F_2_ population. In addition, short deletions on either side of the SNP (S8_5062843) with a total length of 14 nts were detected (Figure 4) and showed the same segregation as the SNP for the sequence of this region on chromosome 8 in both parental lines as given in Table 5.

**TABLE 5:** Nucleotide of the region 5,062,815–506,286 bp in chromosome 8.

NP	CATGTCAAGAGCCATTTACAGGTAGCTATGTATATGCATAGACAGC CTAGACT
KP	CATGTCAAGAGCCATTTA– – – – T– – CT **T** TG– – – – – – – – TAGACAGCCTAGACT

*NP, shattered capsule genotype; KP, non-shattered capsule genotype.*

*Single nucleotide polymorphism is indicated by the italic emphasis. Dash lines indicate deletion.*

**FIGURE 5:**
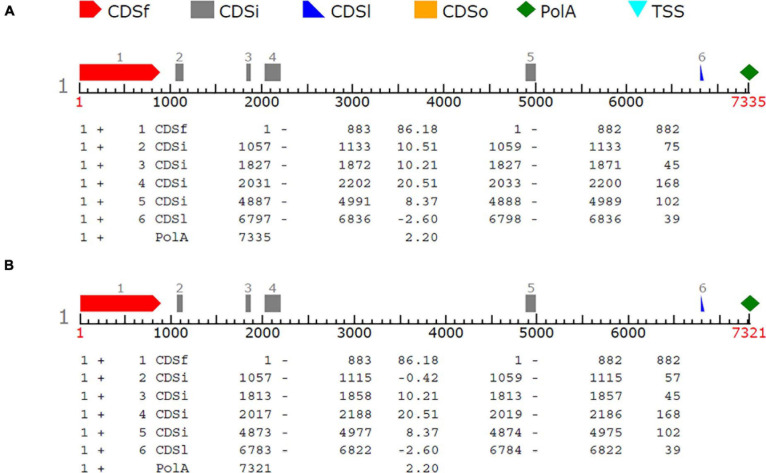
Gene structure of the candidate gene controlling the shattering trait. One gene and six CDSs were predicted in capsule shattering **(A)** and non-shattering **(B)** parents. CDSf, first exon (starting with start codon); CDSi, internal (internal exon); CDSl, last coding segment; PolyA, polyadenylation signal.

In order to reveal genes potentially determining the shattering trait, the SNP region (S8_5062843) was examined with FGENESH software. About 40 kb flanking sequence of the SNP was extracted from the sesame reference genome and analyzed. The results showed that a region covering the SNP marker (S8_5062843) encoded an mRNA including six exons, which was identified as a potential gene related to the shattering trait (data not shown). This sequence region was later amplified with PCR primers, and the sequence of the polymorphic region was confirmed by Sanger sequencing for shattering and non-shattering parents; this produced predicted gene sequences of 6,836 and 6,822 bp in length, respectively. Using tools at the Softberry website to predict gene structure, these sequences were re-analyzed, and six CDSs were predicted in both types (Figure 5). Compared with the reference (capsule shattering) mRNA sequence, the deletions flanking the SNP remove a splice site from the flanking region of S8_5062843, resulting in a shortened exon 2 (Table 5). This altered mRNA is predicted to cause a frameshift mutation starting with the 315th amino acid changing from valine to methionine (Val to Met), which is proposed to result in the loss of function of this gene for the non-shattering parent (Table 6). The potential gene sequence (capsule shattering type) was compared to the non-redundant protein database, and the candidate gene with 440 amino acids shared 99% sequence homology with the predicted transcription repressor KAN1 isoform X1 (*S. indicum*) [XP_011085885.1]. Other KAN1/KAN1-like proteins from different plants (XP_022869422.1, *Olea europaea* var. *sylvestris*; GFP94880.1, *Phtheirospermum japonicum*) also indicated sequence homology > 60% with our sequence. The mRNA sequence of the capsule shattering gene encoding a KAN1/KAN1-like protein has been submitted to NCBI with accession no. MT991091.

**TABLE 6:** Protein sequences of shattering gene for shattered capsule and non-shattered types.

NP	1–MPLEGIFLEPSSKPVPDLSLHISLPNCDSSSSSRSSNTKNDAVSSFDLPVNISSKPKGCT–60
KP	1–MPLEGIFLEPSSKPVPDLSLHISLPNCDSSSSSRSSNTKNDAVSSFDLPVNISSKPKGCT–60
NP	61–SFTDLSLAHPANDEKVELSNSFGRTHQEQEQPQNPYHHHHHRIYQPNHQLNHHQINHGDS–120
KP	61–SFTDLSLAHPANDEKVELSNSFGRTHQEQEQPQNPYHHHHHRIYQPNHQLNHHQINHGDS–120
NP	121–PFDSSDGLRPIKGIPVYPNPPFPFLALDHSTREKDPKMRFYQMSYPSWSSPSSSSSSSSS–180
KP	121–PFDSSDGLRPIKGIPVYPNPPFPFLALDHSTREKDPKMRFYQMSYPSWSSPSSSSSSSSS–180
NP	181–PFFGGGLDHHMPLLNLGPNGSSTAAAYHCGGGGGGGGGGRFSGLSSYQLHHHHNHHHQYG–240
KP	181–PFFGGGLDHHMPLLNLGPNGSSTAAAYHCGGGGGGRFSGLSSYQLHHHHNHHHQYGMGVS–240
NP	241–MGVSHHEGSHHGIMRSRFLPKMPAKRSMRAPRMRWTSTLHARFVHAVELLGGHERATPKS–300
KP	241–MGVSHHEGSHHGIMRSRFLPKMPAKRSMRAPRMRWTSTLHARFVHAVELLGGHERATPKS–300
NP	301–VLELMDVKDLTLAHVKSHLQMYRTVKTTDKPAASSGHSDGSGEDDLSTIGSGSADRTGLR–360
KP	301–VLELMDVKDLTLAHMYRTVKTTDKPAASSGHSDGSGEDDLSTIGSGSADRTGLRQFMEQR–360
NP	361–QFMEQRGPSDVSPRQESDVNNYPAATLWSNSSSSREGSWLQTNAGETSHSLIGSTPFPSQ–420
KP	361–GPSDVSPRQESDVNNYPAATLWSNSSSSREGSWLQTNAGETSHSLIGSTPFPSQSTSGHL–420
NP	421–STSGHLMRKAASKELCNVQF
KP	421–MRKAASKELCNVQF

*NP, shattered capsule parent; KP, non-shattered capsule parent.*

### Marker Development and Validation

In order to differentiate between the capsule shattering and non-shattering genotypes, InDel (S8_5062843) and CAPS (S8_4476867) markers were designed (Table 7). We obtained a single PCR product using the InDel marker, with a size of about 241 bp in shattered genotypes, while the non-shattering genotype produced 227 bp (Supplementary Figure 2) because of deletions in the region of S8_5062843 (Table 5). CAPS markers require a SNP that falls within a restriction site, which is not the case for S8_5062843. However, a nearby SNP positioned at 4,476,867 bp in LG8/chr8 also segregated between the shattering and non-shattering lines, and this polymorphism eliminates a *Taq*I restriction site (Supplementary Figure 3). We designed a CAPS marker for the 4,476,867 C > A substitution that amplified a 377-bp PCR product. This product could not be digested in the non-shattering parent, but it yielded 275- and 102-bp restriction products with *Taq*I enzyme in the capsule shattering parent (Supplementary Figure 2). Heterozygous lines with respect to this SNP also showed both the original PCR product and the digested products (Supplementary Figure 2). These markers were also validated with sesame genotypes (Supplementary Figure 4).

**TABLE 7:** The markers developed to differentiate shattered and non-shattered capsule genotypes.

**Marker type**	**Marker name**	**Forward primer**	**Reverse primer**	**PCR product (bp)**	**Digested products (bp)**
Indel	S8_5062843	AATTTCGATATTTAATTAGGGGCTA	CTGTGCCGGAGAAATAAACG	Shattered type: 241 Non-shattered type: 227	NA
CAPS	S8_4476867	CTGCCTGCTCCCTCAGAGAT	TGCCAATTATTGTACCAAAAAGC	Both types: 377	Shattered type: 102,275 Non-shattered type: 377 (no digestion)

## Discussion

In the present study, we constructed a genetic map in sesame *via* the ddRAD-Seq strategy, which is a powerful SNP diagnostic technique for genotyping. This method produces a large number of sequence tags distributed throughout the genome at restriction sites, allowing a large number of functional polymorphic variants to be identified with low cost (Peterson et al., 2012). It also eliminated random shearing in the library preparation step, leading to high percentages of missing data, which is one of the major shortcomings of the genotyping-by-sequencing (GBS) method (Elshire et al., 2011). Recently, the ddRAD-Seq approach has been used successfully in different plants such as rapeseed (Chen et al., 2017), peanut (Liang et al., 2021), hazelnut (Helmstetter et al., 2020), and lettuce (Seki et al., 2020). However, to our knowledge, this study is the first time that it has been applied in sesame to construct a genetic map. With this technique, we identified 782 SNP markers distributed across 13 LGs; the number of LGs exactly matched the number of chromosomes in *S. indicum* (*n* = 13), showing the effectiveness of the ddRAD-Seq method to identify variants at a genome-wide level. However, large gaps were observed in LG1 and LG7 in our genetic map, and LG11 was only sparsely represented by three polymorphic SNPs (Figure 2). One of the disadvantages of this approach is that occasional genomic regions have very few restriction fragments of the right size to generate markers or may be lacking in polymorphisms. Our results were comparable to those by Shirasawa et al. (2016), who developed genetic maps for tomato with ddRAD-seq, which also left large gaps in some linkage groups. Before the construction of the linkage map, 258 SNP markers, which comprised 24.80% of the polymorphic SNPs, were excluded because of segregation distortion and missing data. Segregation distortion in plants results from gametophyte and/or zygotic selection or chromosomal rearrangements (Menz et al., 2002), and different genetic mechanisms of segregation distortion have been described (Li et al., 2011; Xu et al., 2013). Compared with other studies in sesame, higher values for segregation distortion were obtained by Zhang Y. et al. (2013) and Wu et al. (2014) in high-density genetic map construction. The population type and genetic relationships within the mapping population might also cause differences between gene ratios, leading to segregation distortion (Zhou et al., 2018). The genetic map in the current study covered 697.3 cM, with an average marker interval distance of 0.89 cM. Several genetic maps have been previously reported for sesame, mostly generated using SSR markers or SSRs in combination with other markers. Although these maps had higher length, lower marker densities were observed compared to our result. For example, a sesame genetic map was constructed with EST-SSR, AFLP, and RSAMPL markers mapped into 30 linkage groups covering a genetic length of 936.72 cM; the average distance between markers was 4.93 cM (Wei et al., 2009). A high-density map was constructed using 724 polymorphic markers which were SSR, AFLP, and RSAMPL; these were anchored in 14 linkage groups spanning a total of 1,216.00 cM, with a marker density of 1.86 cM per marker interval (Zhang H. et al., 2013). Another sesame genetic map that incorporated 424 SSR markers and comprised of 13 LGs was reported, with a total length of 1,869.8 cM and a mean density of 5.13 cM (Wang et al., 2017). With the advent of sequencing technologies, comparable high-density maps have been constructed with SNPs in sesame. An F_2_ population was genotyped with SLAF-seq approach, and the resulting map contained 1,233 markers and was 1,474.87 cM in length, with an average marker interval of 1.20 cM (Zhang Y. et al., 2013). This sequencing method was also used by Du et al. (2019) to construct a genetic map of 2,128.51cM in length, with an average distance of 0.99cM between adjacent markers. Wu et al. (2014) developed 224 F_8__:__9_ RILs which were sequenced with RAD-seq, the map, which was 844.46 cM in length, included 1,230 markers with an average of 0.69 cM between adjacent markers. The other reducing representation library sequencing approach, GBS, was employed for genotyping an F_2_ population and constructing a genetic map of 1,086.403 cM in length, with an average of 0.918 cM between adjacent bins (Zhang Y. et al., 2018). Compared to these studies, the total length of the map in our study is a little shorter, and this difference might be a result of the (i) type of mapping population, (ii) number of sequenced genotypes (Li et al., 2017), (iii) sequencing method, and/or (iv) genetic background of parents. Discarded linkage groups and fewer segregation distortion markers in mapping analysis might also be reasons for a shorter map (Wu et al., 2014). Changing the types of restriction enzyme in the library construction or increasing the size of the mapping population might be alternatives to improve this genetic map using the ddRAD-Seq approach (Yang et al., 2020).

The mutant sesame line (PI 599466) had a non-shattering capsule and phenotypically differs from Muganli-57 (shattered capsule cultivar), with curling leaves and oval and smaller capsules at the vegetative stage (Figure 1). These agronomic differences facilitate the selection of genotypes with shattering/non-shattering capsules, especially during the seedling period, in hybridization studies. However, Liu et al. (2020) recently developed a mutant sesame line named JQA that has male infertility and also the curling leaf structure. This showed that the leaf curling observed in genotypes with non-shattering capsule should not be used as a direct phenotypic criterion in early growing stage for the selection of capsule shattering trait. The gene controlling capsule shattering and leaf curling was detected on the eighth chromosome in our study, whereas Liu et al. (2020) found the gene encoding male infertility and leaf shape (curling or rolling) on the 12th chromosome. Different gene pathways in the genome therefore control curling in sesame leaves. Complex developmental processes, including the control of polarity, regulate leaf transverse rolling in plants (Bowman et al., 2002; Micol and Hake, 2003). Studies from different researchers have found that three gene families, KANADI (Eshed et al., 2001), HD-ZIP III (Talbert et al., 1995), and YABBY (Eshed et al., 2004), regulate the establishment of the proximal/distal axis of leaves in *Arabidopsis*. Yan et al. (2008) cloned the SHALLOT-LIKE1 (SLL1)/RL9 gene by way of a map-based cloning strategy and identified that it encodes a GARP protein, an ortholog of the *Arabidopsis* KANADI family in rice. The *sll1* mutant was defective in the regulation of leaf abaxial cell development, resulting in a leaf curling phenotype (Zhang et al., 2009). In our study, the candidate gene controlling the shattering trait was found on chromosome 8, encoding the KAN1/KAN1-like (KANADI) protein that regulates lateral organ polarity and is required for abaxial identity in both leaves and carpels in *Arabidopsis* (Kerstetter et al., 2001). The mutation in that gene is therefore hypothesized to cause loss of cell polarity in developing tissues, resulting in the development of mutants that have non-shattering capsules and curling leaves. Parallel with the current study, a loss of function of the KAN1-like protein was also detected in a curly leaf, non-shattering sesame mutant by Zhang H.Y. et al. (2018), who named this gene SiCl1. The fact that these mutations were discovered independently in the same gene in two independent studies and resulted in the same phenotype confirms that KAN1/SiCl1 determines the non-shattering trait. In *Arabidopsis*, KAN1 was demonstrated to be a transcriptional repressor, which shuts down the expression of several genes involved in tissue development and signaling through the interaction of its Myb-like domain with a 6-bp cis-acting target motif (Huang et al., 2014). Using ChIP-seq and a tiling array, over 200 genes were shown to be directly regulated by *A. thaliana* KAN1 (Merelo et al., 2013). The deletion reported here is at the C-terminal end of the Myb-like domain of sesame KAN1/SiCl1 and therefore likely to result in the loss of its target-binding function; this is also consistent with the recessive nature of the non-shattering trait. It can be hypothesized that, like its ortholog in *A. thaliana*, sesame KAN1 controls leaf patterning, capsule size, and capsule shattering through the regulation of numerous target genes. In future studies, generating mutations in the target genes of sesame KAN1/SiCl1 may enable a discrimination between the non-shattering trait and other undesirable phenotypic effects.

Capsule shattering is one of the most problematic issues in sesame farming because it causes seed losses at harvest of up to 50% (Weiss, 1971). Despite its importance, limited studies have been conducted to improve the understanding of the genomic basis of this trait. In the present study, the gene related to capsule shattering was determined, and new molecular marker technologies were developed. Previously, Uzun et al. (2003) crossed another non-shattering mutant line (cc3) with the Turkish variety Muganli-57, and a closely linked amplified fragment length polymorphism (AFLP) marker was identified in the resulting segregated population. Sequence-characterized amplified region markers associated with shattering resistance were later developed from F_2_ lines derived from a cross between Cplus1, a sesame line with shattering-resistant capsules, and KUAOX25, a line with shattering-susceptible capsules (Phumichai et al., 2017). In the present study, we developed InDel marker related to the shattering trait, and therefore the difference between shattered and non-shattered capsule genotypes can be visualized with basic PCR systems and simple agarose gel electrophoresis. The use of simple genotyping technologies is highly important because sesame breeding is conducted primarily in low-income Asian and African countries. This marker should be integrated into sesame breeding studies with the SSR marker ZMM2498, which determines the growth trait controlled by the dt gene on chromosome 9 (Zhang Y. et al., 2018). In sesame, plant growth is originally indeterminate, and this wildish character causes unwanted agricultural issues such as non-synchronous maturity and incompatibility to combine harvesting (Uzun et al., 2013). Determinate sesame makes a uniform date of maturity, which is one of the requirements for the mechanical harvesting (Uzun and Cagirgan, 2006) of non-shattering capsules. Thus, the markers S8_5062843 and ZMM2498 controlling the shattering and growth habit traits, respectively, can be used as important selection tools in the development of sesame genotypes suitable for mechanized harvesting.

Although non-shattering capsule mutants are theoretically suitable for mechanized harvesting, their low yields currently limit the use of these genotypes in sustainable sesame agriculture. Crossing between mutant and cultivars might be an alternative to obtain heterosis effects for increased yield. This breeding approach about crossing “local varieties × mutants,” “mutants × mutants,” and “mutants × introduced lines” was also suggested to obtain desired traits in sesame (Van Zanten, 2001). In this respect, Uzun et al. (2004) hybridized a non-shattering capsule mutant with local varieties and monitored the heterosis effect in terms of seed yield in all hybrids. In another study, the cultivar Muganli-57 and non-shattering capsule mutant (ACS 344) were introduced into a cross-breeding program, and higher values were acquired for the studied yield traits in capsule shattering types (Yol and Uzun, 2019). Although positive effects were observed on agronomic traits with these crosses, there was no improvement in the shattering trait concerning suitability for mechanized harvesting. Therefore, instead of agronomic approaches, the gene identified in this study should be studied further with gene editing technologies (CRISPR-Cas9, TALEN, and ZFN) to create new variations, which might confer non-shattering capsules without the other negative traits associated with the loss of function of this gene. This strategy was used by Zhai et al. (2019) to understand the functions of pod shattering resistance genes (IND and ALC) using CRISPR/Cas9 technology in rapeseed. A new indica rice line was also developed with the targeted CRISPR/Cas9-mediated editing of the qSH1 gene to obtain lower rates of seed shattering (Sheng et al., 2020). Therefore, the identified gene in the present study should also be assessed with gene editing technologies to make improvements on this trait.

## Data Availability Statement

The datasets presented in this study can be found in online repositories. The names of the repository/repositories and accession number(s) can be found below: https://www.ncbi.nlm.nih.gov/bioproject/PRJNA633276.

## Author Contributions

EY conducted the data analysis, mapping, annotation and wrote the manuscript. MB and SK conducted the F_2_ populations and phenotyping. SL and BU supervised the project and reviewed and revised the manuscript. All authors contributed to the article and approved the submitted version.

## Conflict of Interest

The authors declare that the research was conducted in the absence of any commercial or financial relationships that could be construed as a potential conflict of interest.

## Footnotes

1 www.usegalaxy.eu

2 http://www.softberry.com/berry.phtml?topic=index&group=programs&subgroup=gfind

3 https://primer3.ut.ee/

4 http://nc2.neb.com/NEBcutter2/

## Supplementary Material

Supplementary Figure 1 Total number of reads and GC content (%) for F_2_ lines and two parents.

Supplementary Figure 2 **(a,b)** Amplification of developed InDel (S8_5062843) and CAPS (S8_4476867) markers with selected genotypes, respectively. NP, shattered capsule parent, KP, non-shattered capsule parent. N91, N143, N204, N182, N231, and N258 had shattered capsule phenotypes in F2. K28, K93, and K1 had non-shattered capsule phenotypes in F_2_.

Supplementary Figure 3 IGB shows the **(a)** single-nucleotide polymorphism (SNP) changes in the position of 4476867 on LG8. **(b)** Enzyme digestion with *Taq*I for this SNP. NP, shattered capsule parent; KP, non-shattered capsule parent.

Supplementary Figure 4 **(a,b)** Validation of the developed markers: **(a)** for CAPS marker and **(b)** for InDel marker. NP, shattered capsule parent; KP, non-shattered capsule parent. ACS 169, ACS 195, and ACS 211 were shattered capsule sesame genotypes.
