# Supplementary figures and images for: A High-Density SNP Genetic Map Construction Using ddRAD-Seq and Mapping of Capsule Shattering Trait in Sesame
Source: Front Plant Sci. 2021 Jun 1;12:679659. doi: 10.3389/fpls.2021.679659 (PMC8204047; doi:10.3389/fpls.2021.679659)

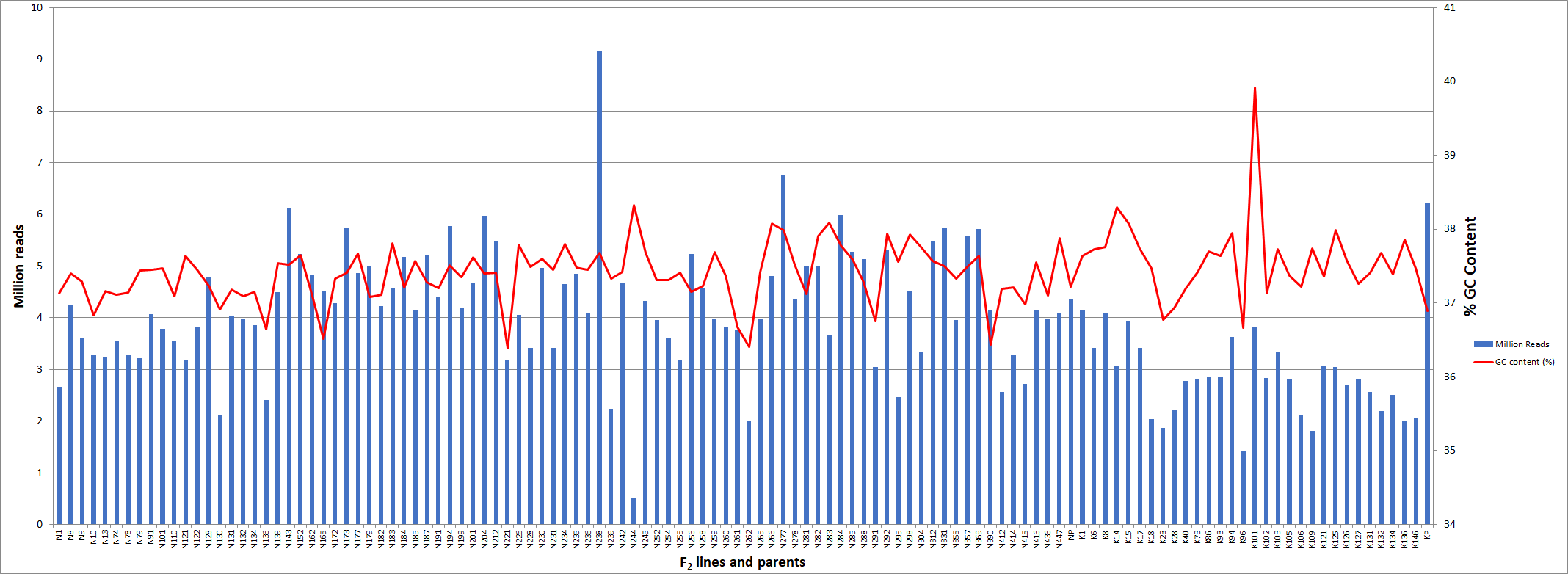

Supplement: Supplementary Figure 1 — Total number of reads and GC content (%) for F2 lines and two parents. [file Image_1.TIF]

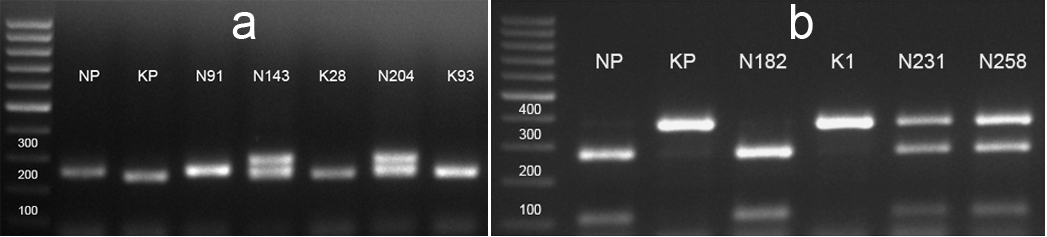

Supplement: Supplementary Figure 2 — (a,b) Amplification of developed InDel (S8_5062843) and CAPS (S8_4476867) markers with selected genotypes, respectively. NP, shattered capsule parent, KP, non-shattered capsule parent. N91, N143, N204, N182, N231, and N258 had shattered capsule phenotypes in F2. K28, K93, and K1 had non-shattered capsule phenotypes in F2. [file Image_2.TIF]

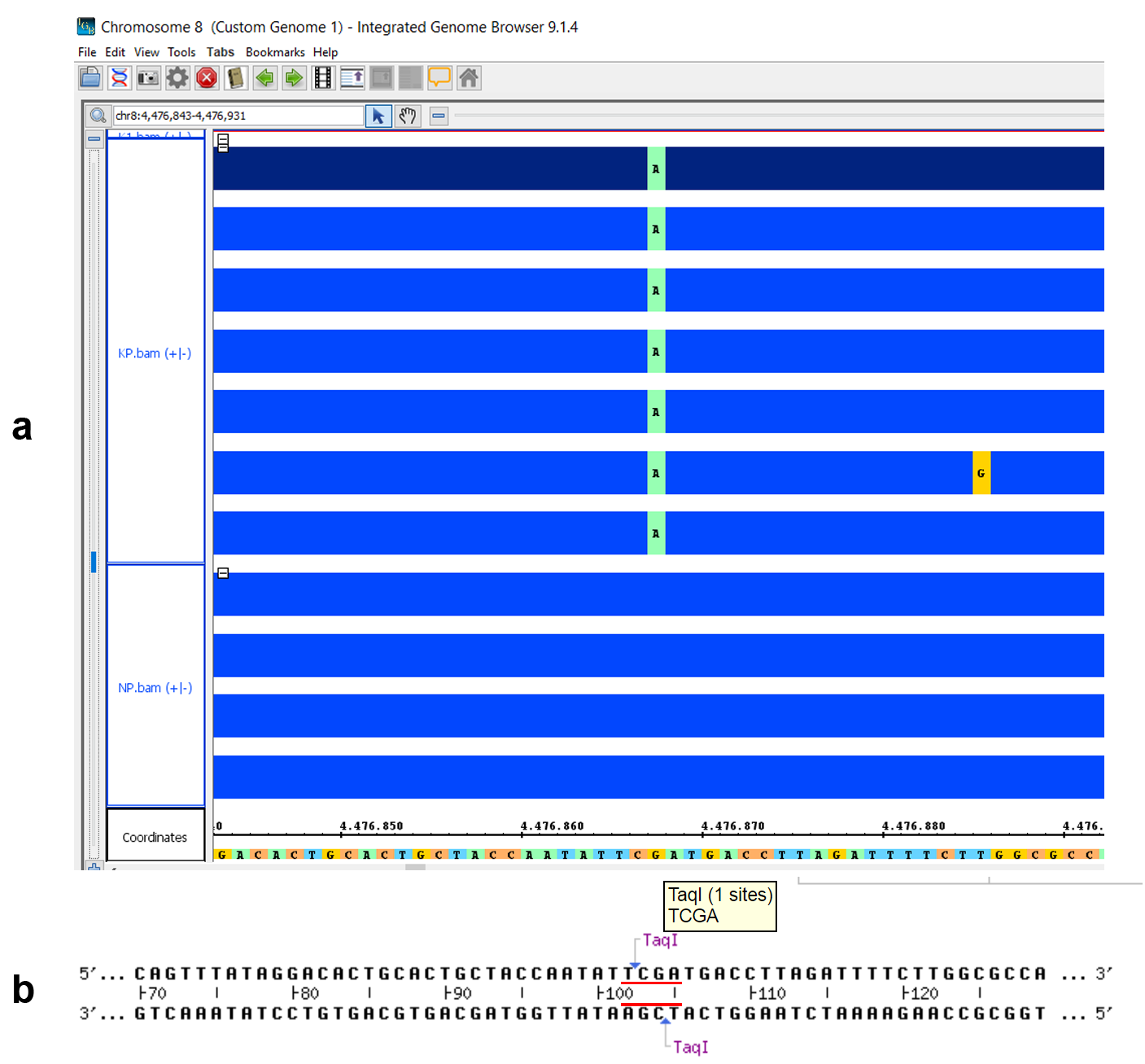

Supplement: Supplementary Figure 3 — IGB shows the (a) single-nucleotide polymorphism (SNP) changes in the position of 4476867 on LG8. (b) Enzyme digestion with TaqI for this SNP. NP, shattered capsule parent; KP, non-shattered capsule parent. [file Image_3.TIF]

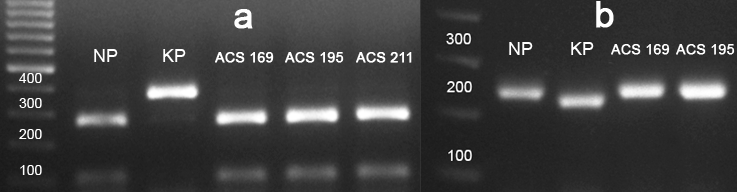

Supplement: Supplementary Figure 4 — (a,b) Validation of the developed markers: (a) for CAPS marker and (b) for InDel marker. NP, shattered capsule parent; KP, non-shattered capsule parent. ACS 169, ACS 195, and ACS 211 were shattered capsule sesame genotypes. [file Image_4.TIF]
